# Editorial: Traditional clinical symptoms and signs: how can they be used to investigate medications in the context of pharmacology?

**DOI:** 10.3389/fphar.2025.1588000

**Published:** 2025-03-31

**Authors:** Kuo Gao, Michael Heinrich, Guang Yang, HuiHui Zhao, XueZhong Zhou, Shao Li

**Affiliations:** ^1^ School of Traditional Chinese Medicine, Beijing University of Chinese Medicine, Beijing, China; ^2^ UCL School of Pharmacy, University College London, London, United Kingdom; ^3^ Chinese Medicine Research and Development Center, China Medical University, Taichung, Taiwan; ^4^ Bioengineering Department and Imperial-X, Imperial College London, London, United Kingdom; ^5^ National Heart and Lung Institute, Imperial College London, London, United Kingdom; ^6^ Cardiovascular Research Centre, Royal Brompton Hospital, London, United Kingdom; ^7^ School of Biomedical Engineering & Imaging Sciences, King’s College London, London, United Kingdom; ^8^ Institute of Ethnic Medicine and Pharmacy, Beijing University of Chinese Medicine, Beijing, China; ^9^ School of Computer Science and Technology, Beijing Jiaotong University, Beijing, China; ^10^ Institute of TCM-X, Tsinghua University, Beijing, China

**Keywords:** clinical symptoms and signs, medication, artificial intelligence, symptom phenotype, image recognition, traditional medicine, network pharmacology, personalized medicine

## Introduction

Modern drug development has traditionally focused on disease pathology, often neglecting the critical role of symptom management. However, symptoms are not merely indicators of disease; they are integral to the patient’s experience and can provide unique insights into therapeutic interventions. In Traditional Chinese Medicine (TCM), symptoms often serve as both diagnostic markers and disease names, bridging the gap between traditional and biomedical systems (Su et al., 2012). This connection suggests that if symptoms are associated with a disease and specific medicines exist for that condition, there may be a latent relationship between the symptoms and the medicines (Bhattacharjee et al., 2024). This relationship is particularly crucial for diseases that are difficult to diagnose or lack effective treatments, even when a diagnosis is clear. Symptom-based treatment aims not only to address the disease but also to improve the patient’s quality of life (Jurgens et al., 2022). However, integrating traditional and biomedical approaches presents significant challenges, as local and traditional disease concepts must be understood within their cultural contexts, and direct correlations with biomedical models are often lacking.

Contemporary pharmacology faces significant challenges in research and development, including high costs, lengthy development cycles, and high failure rates. However, medicines are ultimately meant for human, and their efficacy and effectiveness must be evaluated based on patient responses. Drug development targeting symptoms and signs arises from observations in the real clinical world, which often leads to higher success rates (Liu et al., 2023). Some specific treatments used in TCM, for example, have unique advantages in symptom treatment, with many potential natural medicines waiting to be developed. In particular, TCM practitioners currently prescribe medications based on clinical symptoms and signs rather than solely on diseases (Li et al., 2006). This results in different symptoms and treatments for the same disease due to individual constitution and condition (Sun et al., 2020). This truly embodies a patient-centered approach rather than a disease-centered one. It can be said that symptoms are the targets of TCM treatment. Other medical traditional offer similar opportunities, as exemplified in many studies (Geck et al., 2020).

With advancements in information processing technology, pharmacological research based on symptoms and signs is once more gaining attention. The Research Topic and processing of multi-source big data, including images, are gradually becoming possible, leading to the establishment of relevant databases (Wu et al., 2019). Drug development has brought new opportunities and is expected to open new research fields (Xu et al., 2016).

This Research Topic explores how symptoms and signs facilitate the integration of TCM and biomedicine, featuring contributions from esteemed scholars and garnering positive attention from the academic community. A total of 58 manuscripts from 8 countries and regions were received, and after careful review by editors, reviewers and the Editor-in-Chief including a full assessment using Frontiers’ AIRA (Artificial Intelligence Research Assistant), eleven papers, including ten original articles and one review, authored by 106 scholars were published.

## Clinical diagnosis and individualized treatment in traditional medicine based on symptoms

Personalization is a hallmark of many traditional medical systems including TCM or Korean medicine. Even when patients are prescribed the same herbal medicine, their responses can vary significantly due to individual symptom profiles, such as blood stasis, qi deficiency, qi stagnation, phlegm-dampness, yin deficiency, yang deficiency, and cold congealing. The mechanisms of action of these medicines can differ for the same disease depending on the specific symptoms presentation (Yang et al., 2022). Therefore, both clinical practice and research should emphasize the integration of diseases and symptoms profiles, categorizing symptoms according to their associated diseases (Hansen et al., 2022).

The pharmacology of TCM-based preparations is confronted with challenges and requirements as is the case in many other traditional medical systems. Many studies on the pharmacology of Chinese medicinal plants overlook specific symptoms, resulting in unstable outcomes. Randomized controlled trials (RCT) have important limitations; without appropriate classification under diseases, RCT may be futile (Zhu et al., 2024). TCM emphasizes personalized treatment, tailoring therapies to individual symptom profiles rather than adopting a one-size-fits-all approach. Pharmacological research must clearly define TCM disease classifications, as this is essential for ensuring stable efficacy in treatment (Gao et al., 2017) and a plausible link to a clinical outcome measures of pharmacological models is essential (see the journal’s Four Pillars of Best Practice Section 1b - www.frontiersin.org/files/pdf/4_pillars_FULL_TEXT.pdf). Therefore, whether a specific disease can achieve therapeutic effects depends not only on chemical components but also on the individual’s TCM classification, which is a key insight from this Research Topic’s theme. Of note, there are parallels with some newer and emerging biomedical approaches, where specific efficacy levels are linked to the characteristics of a study population (Snapinn and Jiang, 2007).

Focusing on Kampo medicine, Maeda-Minami et al. developed a machine learning model to predict the four cold–heat patterns in patients exhibiting subjective symptoms as defined in the International Classification of Diseases Traditional Medicine Conditions–Module 1. The identified key items corresponded with the definitions of the cold–heat patterns.

Zedler et al. conducted a study to validate a German-adapted Kampo questionnaire for patients with gastrointestinal diseases, a primary application of Kampo medicine. The study concluded that traditional Kampo questionnaires were valid for analysing a patient’s body constitution (sho) and could guide Kampo treatment. The authors advocated for incorporating individual pattern diagnosis to facilitate personalized treatment approaches.

In another real-world study, Wang et al. used a shared symptom-patient similarity network (PSN) and applying community detection techniques, and they identified distinct subgroups within the Chronic Atrophic Gastritis (CAG) population. This approach offered valuable insights for the classification of CAG and could guide clinical treatment decisions.

He et al. investigated the therapeutic effects of an herbal Chinese preparation - JianPiHuaTan Formula (JPHTF) in patients with colorectal cancer and found significant improvements. The study suggested that JPHTF might inhibit tumour growth in RAS-mutant colorectal cancer by modulating Hippo and Hedgehog pathways, which in turn promoted immune cell infiltration into the tumour microenvironment.

## Multi-omics and data-driven studies of traditional medicine mechanisms and pharmacological targets

Network analysis is an emerging field that utilizes systems biology and bioinformatics to study the complex interactions between medicines and biological systems (Lai et al., 2020; Kong et al., 2024; Liu et al., 2023; Han et al., 2023; Zhang et al., 2023). There are several direct associations among the components including TCM symptom-herb, TCM symptom-modern medicine (MM) symptom, MM symptom–disease, herb–ingredient, ingredient–target and target–disease (also referred to as gene–disease) associations, and other indirect associations for TCM symptom-ingredient, herb-target, ingredient-disease, and MM symptom-target relationships (Wu et al., 2019). Transcriptomics, the study of all RNA molecules in a biological system at a specific time and under certain conditions (Robinson et al., 2022), offers new perspectives in TCM research by uncovering the mechanisms of herbal treatments (Yang et al., 2022). Metabolomics, a systems biology approach, reveals the metabolic state of a biological system by analysing metabolites in biological samples (Ajoolabady et al., 2024; Liu et al., 2025).

Hu et al. conducted a clinical self-controlled trial integrating RNA sequencing and metabolomics to evaluate the effects of Bushen Kangshuai Granules (BKG) on aging in elderly participants. BKG comprises a blend of kidney-tonifying and anti-aging botanical agents - *Panax ginseng* C.A.Mey, *Ophiocordyceps sinensis* (Berk.) G.H.Sung, J.M.Sung, Hywel-Jones and Spatafora [syn.: *Cordyceps sinensis* (Berk.) Sacc.], *Cervus elaphus* L. antler **(** *pilose antler glue)*, *Lycium barbarum* L. berries, and *Cistanche deserticola* Ma herba. Studies have demonstrated that certain botanical constituents and active compounds within BKG exhibit a spectrum of therapeutic effects, mitigating age-related declines in neurogenesis and reproductive function (Shi et al., 2012). The study revealed that BKG significantly reduced levels of superoxide dismutase (SOD) and TCM aging symptoms. The PI3K-AKT signalling pathway and sphingolipid metabolism might be potential mechanisms underlying its anti-aging effects.

Based on high throughput mRNA sequencing, Li et al. identified the pons in the brainstem as a critical brain region mediating the antidepressant effects of Si-Ni-San (SNS). SNS is a TCM herbal formula, originating from the foundational TCM text Shang Han Lun (Treatise on Febrile Diseases). This formulation comprises four key botanical components in equal proportions (1:1:1:1): Bupleuri radix (*Bupleurum chinense* DC.), Paeoniae radix alba (*Paeonia lactiflora* Pall.), Aurantii fructus immaturus (*Citrus trifoliata L.*), and Glycyrrhizae radix et rhizoma praeparata cum melle (*Glycyrrhiza uralensis* Fisch.). In clinical TCM practice, SNS has demonstrated considerable potential in the management of major depressive disorder. Their study demonstrated that SNS enhances pontine norepinephrine levels through the regulation of key genes, including Anxa1, Nrg1, and Psen1.

Wang et al. conducted a comprehensive study leveraging data from the SymMap and MalaCards databases, combined with real-world clinical data and experimental evaluation, to investigate the molecular mechanisms underlying the effects of a preparation based on *H. diffusa* Willd. (a synonym of *Scleromitrion diffusum* (Willd.) R.J.Wang [Rubiaceae]) in the treatment of lung adenocarcinoma (LUAD). The results demonstrated that *Hedyotis diffusa* inhibited the proliferation and promoted apoptosis in LUAD A549 cells. These effects were found to be associated with the regulation of CTNNB1 expression.

Zhang et al. employed LC-MS/MS chemical analysis combined with a systems biology approach to investigate the antidepressant effects and underlying mechanisms of the ethyl acetate extract (ECS) of *Cynomorium songaricum* Rupr. *In vivo*, ECS was shown to reduce the expression of inflammatory factors in the hippocampus, inhibit M1 microglial cell polarization, and alleviate depressive symptoms through modulation of the NF-κB-NLRP3 inflammasome pathway.

## Exploring molecular mechanisms of traditional medicinal plants based on animal and cell models

In TCM research, animal and cell models are extensively used to evaluate the pharmacological effects and safety of Chinese medicines. These models allow researchers to explore the mechanisms of action of herbal treatments, providing scientific evidence for drug development (Yan et al., 2025).

Zhachong Shisanwei Pills (ZSP), a highly esteemed Mongolian medicinal formulation, comprises thirteen traditional herbal ingredients and is used to ease tension in the tendons, enhance blood circulation, and impart a calming and tranquilizing effect on the mind. As a result, it is extensively employed by the Mongolian population in China as an adjunctive therapy in the management of ischemic stroke. Hu et al. demonstrated that ZSP exerted protective effects against H_2_O_2_-induced oxidative stress and apoptosis in PC12 cells. The underlying mechanism appears to involve the inhibition of the MAPK signalling pathway, enhancement of antioxidant enzyme activity, reduction of intracellular peroxidation levels, and suppression of intrinsic apoptosis pathways.

Bushen Huoxue formula (BHF), a traditional clinical prescription, consisting of eleven medicinal plants used in kidney-tonifying and activating blood, is widely used in China for ovarian failure-related diseases. Li et al. provided compelling evidence for the therapeutic potential of BHF in treating premature ovarian insufficiency (POI). Their findings suggested that BHF alleviated POI symptoms by modulating lipid metabolism, thereby reducing lipid accumulation-induced reactive oxygen species and autophagy.

In a comprehensive review, Zheng et al. highlighted the potential of metabolites found in TCM preparations in mitigating ischemia and reperfusion injury. These metabolites promoted neurological recovery through modulation of key mechanisms, including oxidative stress, inflammation, programmed cell death, glutamate excitotoxicity, and calcium overload.

## Conclusion

**FIGURE 1:**
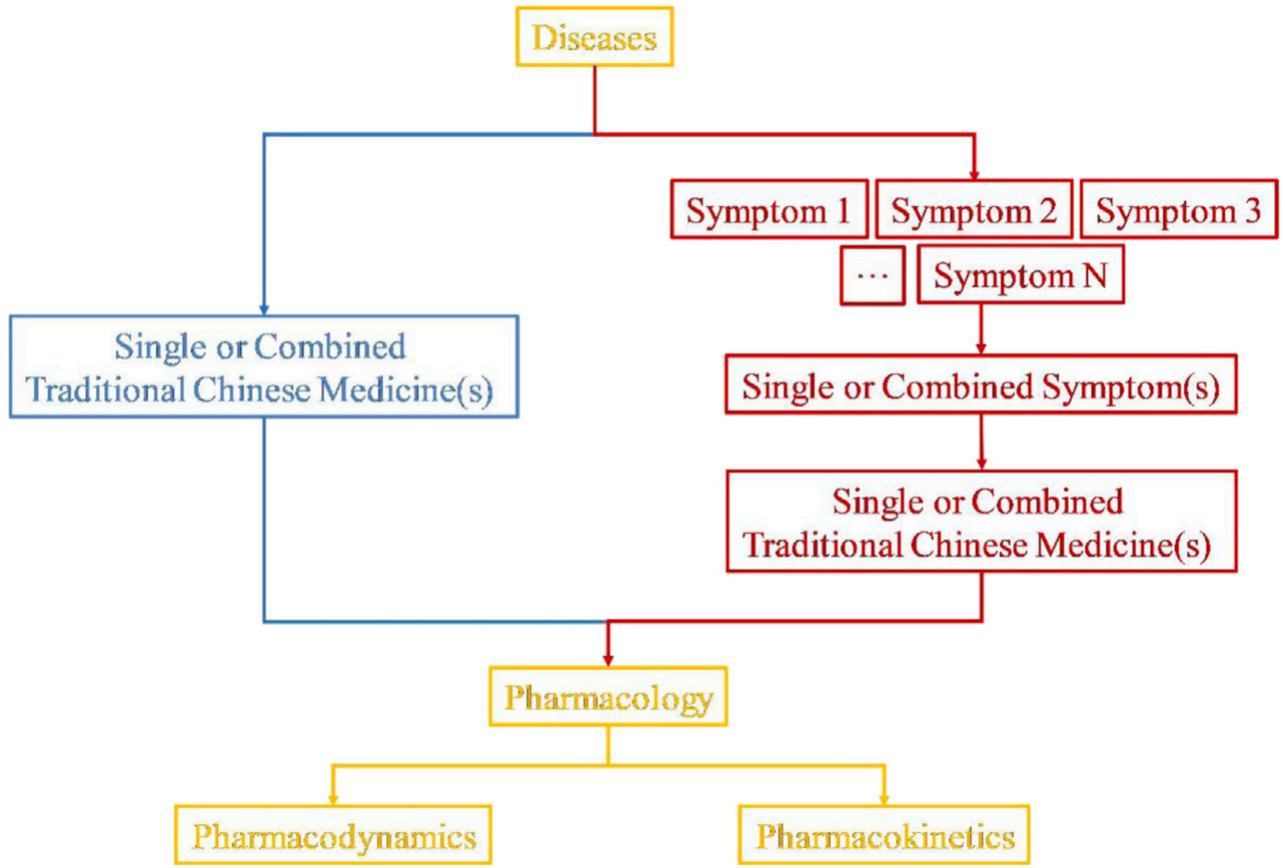
The integration of disease and symptom profiles serves as a fundamental principle in pharmacological research within Traditional Chinese Medicine (TCM), providing a valuable framework for guiding personalized treatment strategies. This approach firstly categorizes a single disease based on distinct symptomatic manifestations and subsequently investigates the pharmacological mechanisms of the corresponding therapeutic TCMs.

This Research Topic highlights the significance of symptom-oriented drug development, demonstrating how the integration of traditional and modern pharmacological approaches can lead to more effective and personalized treatments (Figure 1). By incorporating animal and cellular experiments, researchers can further explore the biological foundations of symptom-targeted therapies in traditional medical systems such as Kampo or TCM. Several studies demonstrated the potential of methodologies focused on understanding symptoms, while others have adopted a more traditional approach based on pharmacological mechanisms. These emerging methodologies may enable the identification of novel therapeutic applications for existing pharmacologic agents, elucidate latent therapeutic associations, and refine drug development strategies, thereby advancing innovative directions in clinical pharmacology.
